# Why the ‘V’ matters: advancing equity and effectiveness in violence against women interventions through trauma and violence informed care

**DOI:** 10.3389/frph.2026.1770836

**Published:** 2026-03-19

**Authors:** Tara Mantler, C. Nadine Wathen

**Affiliations:** 1 Faculty of Health Sciences, School of Health Studies, Western University, London, ON, Canada; 2 Faculty of Health Sciences, Arthur Labatt Family School of Nursing, Western University, London, ON, Canada

**Keywords:** equity, health, SRHR, trauma and violence informed care, violence

## Abstract

Intimate Partner Violence (IPV) is a pervasive global health and human rights wherein experiences are shaped by intersecting social positions. Despite the complex manifestations of violence, health-sector responses often prioritize acute crises while neglecting the structural forces that perpetuate it. Trauma- and Violence-Informed Care (TVIC) offers a transformative, equity-oriented approach bridging clinical practice with social change by explicitly acknowledging the role of systemic oppression, institutional violence, and inequitable hierarchies that shape access to care. By expanding trauma-informed practice to incorporate an understanding of structural and interpersonal violence, TVIC reframes trauma as both an individual and collective experience rooted in social and structural determinants of health. The “V” in TVIC highlights the need to address systemic harms that position some survivors as more deserving of care than others, perpetuating cycles of revictimization. TVIC calls for multi-level action: fostering compassionate, non-judgmental, strengths-based care at the individual level; embedding culturally, emotionally, and physically safe organizational practices; and promoting intersectoral collaboration to reduce systemic barriers. In SRHR contexts, TVIC restores dignity and autonomy by challenging entrenched inequities and mitigating risks of retraumatization. Adopting TVIC offers an equity promoting, tangible way to optimize systems of care and finally interrupt violence cycles of oppression.

## Introduction

Our previous work in Intimate Partner Violence (IPV) and equity has been focused on providing practical guidance in ways to integrate TVIC thereby bridging clinical practice and social change, affirming that health care is inseparable from human rights. IPV is a well-established and pervasive global health problem and human rights issue that disproportionately affects women, girls, and equity-denied people and groups. Experiences of violence are mediated by intersecting social positions including based on gender, racialization, Indigeneity, class, sexuality, disability, and immigration status. Among health sector actors, IPV is often understood as physical violence and resultant injuries with various health consequences. However, IPV takes many forms, including physical, psychological, and sexual violence, as well coercive control including isolation, harassment and financial abuse, and often co-occurs and is causally linked to other forms of structural violence and oppression. Health care responses to IPV often prioritize those in acute crisis, neglecting its chronic effects, as well as its “root causes” including patriarchal gender norms and barriers to the social and structural determinants of health. To account for these complexities to IPV recognition and response at both social/systemic, and family/individual levels, we have proposed Trauma- and Violence-Informed Care (TVIC) as a transformative framework that integrates trauma theory with specific attention to the harms of systemic violence and social inequities embedded in the systems designed to help survivors (1).

### The importance of the ‘V’

Systems and structures operate on, and within, implicit hierarchies that determine who is advantaged and who is disadvantaged. These hierarchies influence resource allocation and create barriers for those with intersecting vulnerabilities such as poverty, housing insecurity, racialization, colonization, etc. When survivors of violence seek care, these systemic forms of oppression are operationalized into implicit judgements (through intake policies, etc.) that position some people as deserving of care, and others as not deserving of care. Combined with implicit biases that may also be operating at the intra- and interpersonal levels, a cycle of (re)victimization is created/perpetuated, rooted in the survivor's experience of IPV, but then amplified through these oppressive systems and practices, often resulting in victim-blaming, denial of needed services and supports, and survivors feeling punished for daring to ask for help. While these patterns of harm are especially prevalent for survivors of sexual violence in the criminal-justice system, they are also pervasive in health care, child welfare and other social service systems.

### Understanding the ‘V’

TVIC builds upon prior iterations of trauma-informed practice (TIP) (2–9) by explicitly acknowledging the role of structural and interpersonal violence in shaping health and social outcomes. TIP emphasizes safety, choice, and collaboration, and focuses on strategies to avoid reactivating trauma in care interactions. TVIC adds an understanding of, and specific focus on, intersecting forms of oppression, discrimination and stigma. Adding the ‘V’ reframes trauma as not only an individual experience but also a collective reality of the inequitable way that systems oppress people from equity denied groups. This shift moves the focus at the service level from “what steps can we take to ensure an individual is safe when accessing our services?” to ‘how can we adopt practices to reduce the risk of re-traumatization and address the effects of systemic, institutional and interpersonal violence and oppression for those we serve?”. This systems approach acknowledges and aims to dismantle barriers that often unintentionally harm service users.

The work of the ‘V’ is to make structural violence and systemic oppression explicit and to neutralize inequitable practices and policies through multi-level interventions (i.e., individual, organizational, and systemic). At the individual level, we can provide care based on the assumption of histories of trauma and violence, with knowledge about how these might present, and how to respond, without judgement and with compassion and respect, building on strengths and fostering agency and choice. At the organization level, we can embed physically, emotionally and culturally safe practices and focus on identifying where policies and protocols apply oppressive hierarchies, reworking them using an equity lens. At the systemic level, we can find ways to support interagency and intersectoral collaboration such that our systems become designed to support those with complex trauma, where any door is the right door to good care. TVIC underscores the reality that healing and safety cannot occur in isolation from the social and structural determinants of health. TVIC reframes care as both clinical and political, a necessary shift to meaningfully address the consequences of violence.

### Equity and the ‘V’

Equity must be foundational, not secondary, in supporting those experiencing violence. Systems of care are often funded and operate on a one-size-fits-all approach, ignoring the cumulative and often compounding effect of intersectional identities and how these are positioned in oppressive hierarchies. TVIC embeds equity by guiding care interactions, environments, and policies attending to those experiencing the harms of these oppressive hierarchies. It focuses on supporting direct service providers in the 'sticky' points of practice, where policy interferes with the ability to do ‘good’ work, by examining not only individual provider responses but also how systems are designed to respond to survivors of violence. Taking a TVIC approach not only improves the acceptability of services, but also decreases barriers for those using services, and supports direct service providers in instances where equitable care and organizational policy collide.

### Sexual and reproductive health and human rights (SRHR) and the power of the ‘V’

In SRHR contexts, TVIC restores autonomy and dignity. Sexual and reproductive care has strong potential for (re)traumatization due to its intimate nature, and the politicization of women's bodily autonomy. Some victim-survivors are deemed more “believable” and “worthy” than others, by both individual providers, and by practice systems and protocols that may still be grounded in oppressive hierarchies. Some forms of interpersonal and systemic harm, such as reproductive coercion and obstetric violence, are not well-understood or even recognized (10, 11). This has implications for how care and system navigation are experienced, and indeed on who will even seek or receive appropriate care in the first place. By focusing on creating safe environments and challenging policies and systems that disadvantage certain survivors, we can work toward dismantling systemic and implicit biases that perpetuate harm and inequities. This involves embedding practices that prioritize respect, trust, transparency, and safety, while actively challenging organizational and policy-level barriers that work to undermine survivor credibility. By doing so, SRHR services can move beyond a one-size-fits-all approach and create care environments that affirm autonomy, dignity, and equitable access for all people, regardless of their intersecting social positioning or experiences of violence.

Given evidence that critically and theoretically oriented interventions are emerging as those with the most promise of benefit, both in supporting survivors (1) and in preventing (re)use of violence in relationships (12), we argue that strengthening SRHR responses to survivors requires adopting TVIC as a foundation for practice and policy. Interventions, policies and protocols need to be equity-driven, responsive to structural barriers, and work to confront the root causes of violence, especially patriarchy. Building on our previous work to provide practical guidance in the area of violence against women and ways of integrating TVIC, TVIC is a call to action that offers an equity promoting, tangible way to optimize systems of care and finally interrupt violence cycles of oppression.

## Data Availability

The original contributions presented in the study are included in the article/Supplementary Material, further inquiries can be directed to the corresponding author.
